# Pharmacological Modulation of Blood–Brain Barrier Permeability by Kinin Analogs in Normal and Pathologic Conditions

**DOI:** 10.3390/ph13100279

**Published:** 2020-09-29

**Authors:** Dina Sikpa, Lisa Whittingstall, Martin Savard, Réjean Lebel, Jérôme Côté, Stephen McManus, Sylvain Chemtob, David Fortin, Martin Lepage, Fernand Gobeil

**Affiliations:** 1 Department of Nuclear Medicine & Radiobiology, Université de Sherbrooke, Sherbrooke, QC J1H 5N4, Canada; Dina.Sikpa@USherbrooke.ca (D.S.); Lisa.Whittingstall@Usherbrooke.ca (L.W.); Rejean.Lebel@USherbrooke.ca (R.L.); 2 Department of Pharmacology & Physiology, Université de Sherbrooke, Sherbrooke, QC J1H 5N4, Canada; Martin.Savard@USherbrooke.ca (M.S.); jerome.cote@usherbrooke.ca (J.C.); stephen.mc.manus@usherbrooke.ca (S.M.); 3 Department of Pharmacology & Physiology, Université de Montréal, Montréal, QC H2X 0A9, Canada; Sylvain.Chemtob@UMontreal.ca; 4 Department of Surgery, Université de Sherbrooke, Sherbrooke, QC J1H 5N4, Canada; David.Fortin@USherbrooke.ca

**Keywords:** kinin analogs, G protein-coupled receptors, blood–brain barrier permeability, chemotherapy, radiotherapy, CNS diseases

## Abstract

The blood–brain barrier (BBB) is a major obstacle to the development of effective diagnostics and therapeutics for brain cancers and other central nervous system diseases. Peptide agonist analogs of kinin B1 and B2 receptors, acting as BBB permeabilizers, have been utilized to overcome this barrier. The purpose of the study was to provide new insights for the potential utility of kinin analogs as brain drug delivery adjuvants. In vivo imaging studies were conducted in various animal models (primary/secondary brain cancers, late radiation-induced brain injury) to quantify BBB permeability in response to kinin agonist administrations. Results showed that kinin B1 (B1R) and B2 receptors (B2R) agonists increase the BBB penetration of chemotherapeutic doxorubicin to glioma sites, with additive effects when applied in combination. B2R agonist also enabled extravasation of high-molecular-weight fluorescent dextrans (155 kDa and 2 MDa) in brains of normal mice. Moreover, a systemic single dose of B2R agonist did not increase the incidence of metastatic brain tumors originating from circulating breast cancer cells. Lastly, B2R agonist promoted the selective delivery of co-injected diagnostic MRI agent Magnevist in irradiated brain areas, depicting increased vascular B2R expression. Altogether, our findings suggest additional evidence for using kinin analogs to facilitate specific access of drugs to the brain.

## 1. Introduction

A major issue in treating brain cancers and other central nervous system (CNS) diseases is drug access to specific areas of the brain. This is mostly due to the presence of the blood–brain barrier (BBB) [1,2,3,4,5,6]. Most approaches to delivering drugs across the BBB have, thus far, consistently failed to meet regulatory approval due to a variety of significant disadvantages. Current approaches under development include convection-enhanced delivery (CED), focus-ultrasound (FUS), cerebral intra-arterial infusion with and without osmotic BBB disruption, and molecular Trojan horse-related methods. These approaches are either highly invasive (e.g., CED), require specialized equipment (e.g., FUS), or necessitate the conjugation of drugs to specific targeting-peptide vectors in limited stoichiometric ratios (e.g., Angiopep-2). For drug delivery to brain cancers, osmotic BBB disruption with mannitol is the only effective method that has successfully made its way into the clinic, with nine International BBB-Consortium Centers operating across the United States, Israel, and Canada, including in our *Centre hospitalier universitaire de Sherbrooke* (CHUS, Québec, Canada) [6,7,8]. However, duly approved, it remains highly invasive, requires general anesthesia, and is accompanied by the nonselective delivery of anticancer drugs to normal brain tissues, and is thus not compatible with chemotherapeutics that are neurotoxic (e.g., taxanes and cisplatin) [9].

Kinins are labile, short linear peptides (8 to 10 amino acids) that drive brain vascular blood flow and permeability. They are also potent enhancers of the desirable enhanced permeability and retention (EPR) effect in tumors [10,11,12]. Kinin effects are mediated through specific activation of two types of receptors, namely, the kinin B1 (B1R) and B2 receptors (B2R), both belonging to the G-protein-coupled receptor family. The biostable peptide B2R agonist Labradimil, formerly referred to as RMP-7 and Cereport, has previously been shown to induce transient permeation of the BBB, thereby improving intracerebral delivery and effectiveness of numerous drugs, such as antineoplastic, anti-Parkinson, analgesic, and antiviral agents in different preclinical models of CNS diseases [2,13]. This bradykinin (BK) analog has also been tested as adjuvant intravenous therapy in combination with carboplatin in a few clinical trials for the treatment of primary/recurrent brain cancers with mixed results [14,15,16,17]. Along this line, our group has shown that biostable B1R or B2R agonists promote the selective delivery of co-injected diagnostic MRI agents (e.g., Magnevist (0.5 kDa) and Gadomer (17 kDa)) or the chemotherapeutic drug carboplatin (0.4 kDa) across the BBB, within experimental malignant brain tumors (i.e., F98-Fischer syngeneic rat glioma model) [18,19,20]. Recently, we provided evidence that dual activation of kinin B1R and B2R on the BBB by use of selective agonists, either in monomeric or dimeric form, may produce additive effects in terms of enhancing brain cancer delivery and efficacy of intraarterial carboplatin, translating into prolonged animal survival [18]. In light of emerging studies highlighting the potential of exogenous monocytes/macrophages as nanomedicinal delivery systems for treating inflammatory and neurodegenerative diseases, another noteworthy aspect for the possible application of BK analogs is that they can facilitate the transfer of intact, viable monocytes across the BBB into the CNS at neuroinflammation sites [21,22,23,24]. These findings highlight how modulating BBB permeability in vivo with vasoactive stabilized kinin analogs could enhance the brain bioavailability and efficacy of CNS diagnostics and therapeutics, including anticancer drugs and immune cell-based drug delivery systems.

The main purpose of the study was to further expand the knowledge of the ability of biostable kinin analogs to modulate BBB permeability in normal and pathologic conditions (i.e., primary/secondary brain cancers, late cerebral radiation necrosis). More specifically, this study is intended to provide some answers to questions, such as (1) could kinin analogs be useful to enhance brain tumor delivery of another class of chemotherapeutics, namely doxorubicin (DOX); (2) is there a size dependency of the BBB opening induced by B2R agonists; (3) could the transient pharmacological opening of the BBB by B2R agonists lead to increased formation of breast cancer brain metastases; and 4) what is the long-term impact of prior brain radiotherapy on the kinin B2R-mediated increases in BBB permeability. For the proposed in vivo investigations in mice, we selected the peptide [Hyp^3^, Thi^5^, ^N^Chg^7^, Thi^8^]-BK (NG291) as a selective surrogate agonist for the B2R. This B2R agonist exhibits high affinity and potency in vitro and efficacy in vivo [25,26], with demonstrated therapeutic properties in experimental cardiac, peripheral ischemic, and diabetic diseases [27,28,29]. Moreover, like Labradimil, it is fully protected against enzymatic degradation by the angiotensin-converting enzyme (ACE, alias kininase II), the primary contributor to BK inactivation in the blood and in the vasculature [26].

## 2. Results

### 2.1. Comparative Evaluation of Binding Affinities and Functional Activities of BK Analogs at Human B2R

Table 1 shows a comparative pharmacological evaluation of BK and four related agonist analogs, among which three of them have been preclinically tested as BBB permeabilizers (NG291, Labradimil, RI-BK) for glioma treatments. The molecular masses and purities (exceeding 95%) of the synthetized peptides were formally validated prior to being used for binding and functional assays using living adherent HEK293 cells stably expressing human B2R (Table 1). As it is known that B2R is mostly coupled to the Gαq pathway, the functional assays consisted of testing the ability of BK and its analogs to stimulate the inositol monophosphate (IP) cascade by measuring IP1 accumulation over 30 min. Results indicated that the peptide NG291, like BK, displayed higher binding affinity (IC_50_) and greater potency (EC_50_) values than Labradimil, in accordance with our previous in vitro findings [25]. By comparison to NG291, the peptide agonist B9972 exhibited 150–200 fold less affinity/activity, albeit it could theoretically be more peptidase-resistant than NG291 owing to protection at its N- or C-termini. In our hands, RI-BK demonstrated no binding affinity and agonist activity at human B2R. Similar negative results were obtained with RI-BK using ex vivo contractility bioassays based on the isolated human umbilical vein, which expresses endogenous B2R [25] (data not shown). Altogether, the findings validate the peptide NG291 as an appropriate pharmacological tool for assessing the in vivo B2R-mediated modulation of BBB permeability to enhance drug delivery.

### 2.2. Effects of Kinin B1 and B2 Agonist Analogs on DOX Delivery to Tumor in F98 Glioma-Bearing Rats

**Figure 1:**
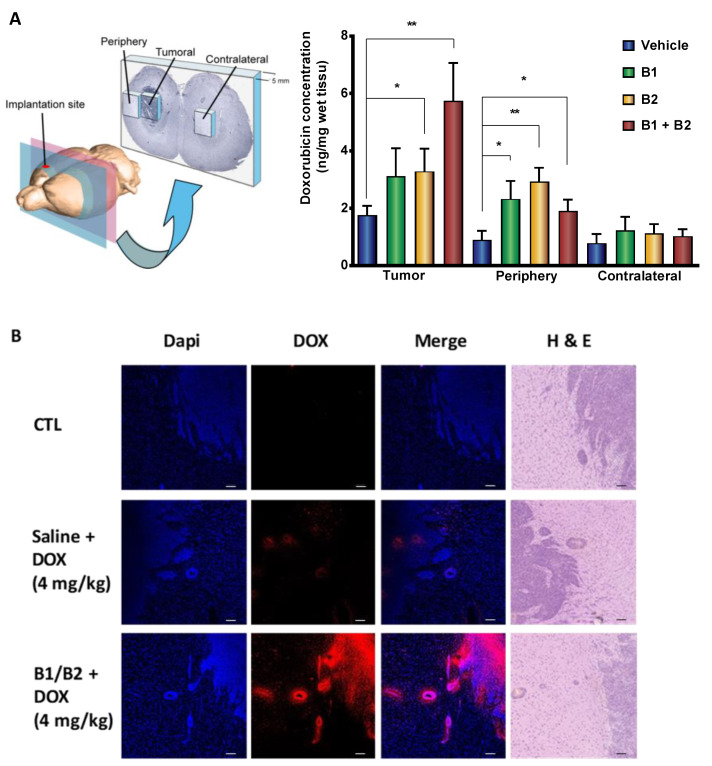
Intracarotid administration of synthetic kinin B1 and/or B2 receptor agonists selectively enhances doxorubicin (DOX) delivery to tumors in F98 glioma-bearing rats. (**A**) Histographic representation with 3D schematic drawing of dissection of rat brain (left panel) and spectrofluorometric quantification of DOX (Ex/Em: 480/590 nm) in sized-related samples from tumoral, peritumoral, and matched tissues from the contralateral hemisphere (right panel). Data are reported as means ± SEM for *n* = 7–9 animals per group. * *p* < 0.05, ** *p* < 0.01 vs. vehicle-treated group, Student’s unpaired *t*-test (GraphPad Prism 8.2). (**B**) Imaging of DOX distribution in rat tumor/peritumoral sites by confocal microscopy. Representative confocal midsection images of brain tumor sections of F98 glioma-bearing rats left untreated or treated with DOX solely or combined with B1 receptor (B1R) and B2R agonists (*n* = 2 rats/group; qualitative data). Contiguous sections stained with Dapi (to identify cell nuclei) and H and E are shown. Scale bar = 150 µm.

To substantiate our previously reported results [18,19,20] and further support the utility of kinin receptor agonist analogs to increase the penetration of chemotherapeutic drugs to tumors, we conducted additional testing with the chemotherapeutic DOX (0.5 kDa). We choose to use DOX for several reasons: (1) it is more potent than temozolomide (TMZ) for killing GBM cells in vitro; (2) it is widely used against many cancer types but remains ineffective in vivo against brain cancer; (3) it does not readily cross the BBB and so shows poor efficacy in in vivo models; and (4) it is an intrinsically fluorescent agent that can be quantified by fluorometric analysis [33]. The results showed that intracarotid (i.c.) B1R and B2R agonists significantly enhanced the uptake of DOX in tumor and/or surrounding normal brain tissues in F98 glioma-bearing rats compared to the vehicle-treated group (Figure 1A). Such increases in DOX levels were not observed in equivalent regions of contralateral brain tissues. Notably, the combination of B1R and B2R agonists caused a further increase in the uptake of DOX in tumors, suggesting an additive effect of the agonists. These latter results were confirmed by fluorescence confocal microscopy. As can be seen in Figure 1B, the implementation of the blood–tumor barrier (BTB) opening procedure with the B1R/B2R agonist combo before DOX administration resulted in higher fluorescent DOX distribution at primary sites and perivascular satellite tumor nodules. The increased drug delivery at peritumoral sites afforded by kinin agonist co-administration is relevant, as infiltrative glioma cells left behind after surgery and local therapy are likely responsible for tumor recurrence.

### 2.3. Effects of Kinin B2R Agonist Analog NG291 on the BBB Permeability in Normal Condition

**Figure 2:**
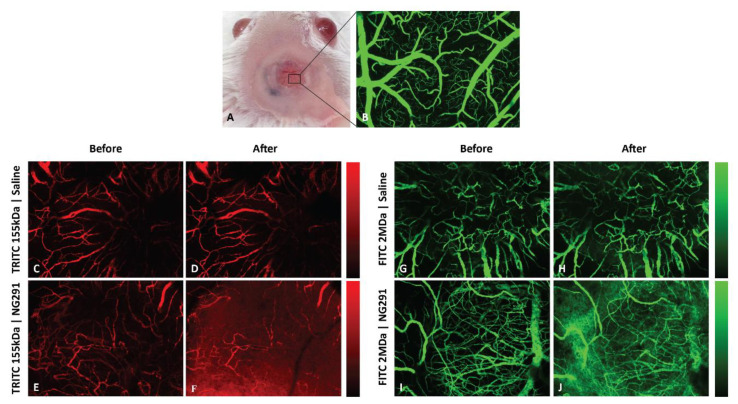
NG291 increases blood–brain barrier (BBB) permeability to large fluorescent dextran tracers in normal mice. (**A**) Image of a craniotomy over a mouse right cortex. Superficial blood vessels are clearly discernable through the glass window. (**B**) Maximum intensity z projection image (through the depth of 0–518 µm) from intravital confocal microscopy of a region under the cranial window in A showing the vascular network after intravenous injection of fluorescein isothiocyanate (FITC)-dextran (2 MDa). (**C**–**J**) Representative intravital confocal Z-stack images of mouse brain microvasculature (pial vessels) following i.v. injection of (**C**–**F**) tetramethylrhodamine (TRITC)-dextran (mw 155 kDa; Stoke’s radius ~ 8.5 nm) or (**G**–**J**) FITC-dextran (mw 2 MDa; Stoke’s radius ~ 27 nm) (**C**,**E**,**G**,**I**) before and 30 min post-injection of (**D**,**H**) saline or (**F**,**J**) the B2R agonist NG291 (50 µg/kg or 1.25 µg/mouse). Pseudo-color bars show fluorescence intensity scales ranging from black (no intensity) to red (high intensity). Qualitative differences in fluorescence of 155 kDa or 2 MDa dextrans between saline and NG291-treated animals are depicted (*n* = 3 mice/group). Dextran leakage usually occurred 60 s after NG291 injection. Injection of the vehicle did not affect BBB permeability.

To capture in vivo images of the effects of kinin B2R agonist on healthy, noncompromised BBB permeability, we used intravital microscopy via an open-skull cranial window with a glass cover to allow the qualitative direct visualization of cerebrovascular changes to a defined cortical area in real-time (Figure 2A). To visualize the cortical vascular network and assess BBB integrity, fluorescently labeled dextrans with molecular weights of 155 kDa and 2 MDa (hydrodynamic diameter: ~17 to 54 nm, respectively) were first delivered via intravenous (i.v.) injection. These were used respectively as models of monoclonal antibodies (mAbs) and nanoparticles (NPs)-based drug formulations, both of which are currently viewed as very promising approaches in the fight against malignant brain cancers [34]. No spontaneous microvascular leakage was observed immediately after injection of the fluorescent tracers (Figure 2B,C,E,G,I), indicative of a preserved BBB integrity. Next, sequential imaging was performed to monitor the leakage of labeled dextran for 30 min after the injection of either vehicle saline or NG291 (Figure 2C–J). While the control group exhibited no significant change (Figure 2D,H), we observed clear leakage of both fluorescent dyes into the perivascular area after NG291 injection (50 μg/kg, bolus injection, see also Supplementary Videos S1 and S2). The extent of leakage appeared to be more important for the smaller (155 kDa) than for the larger dye (2 MDa) (Figure 2F,J). Although generally well-tolerated, the high dose of intravenous NG291 was associated with a strong but reversible hypotensive effect (about -35 mmHg) in ketamine/xylazine-anesthetized normal mice (not shown). These findings are in agreement with previous studies [18,26,27,28].

### 2.4. Effects of Acute Administration of the Kinin B2 Agonist Analog NG291 on Brain Metastases

**Figure 3:**
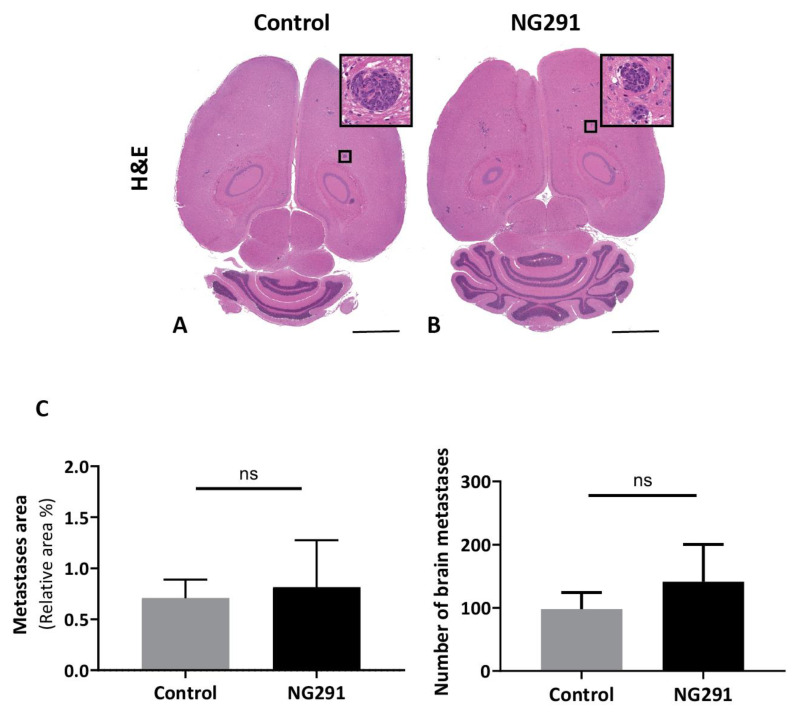
Effects of acute administration of NG291 on breast cancer dissemination to the brain. Quantification of metastases area on histological brain slides 18 days following intracardiac injection of 4T1 cells. The control group received only an intracardiac injection of 4T1 cells. In the NG291 group, 4T1 cells were injected with the B2R agonist. (**A**,**B**) Representative H and E staining of brain sections from animals in the aforementioned groups (magnification 1×; scale bar: 2 mm). Insets are high-resolution images (40× magnification) of the area in the black box. (**C**) Quantification of surface area and number of cerebral metastases. Results are presented as the percentage of metastasis area relative to the entire brain section. There was no difference in terms of both area and number of metastases between the group injected with NG291 and the group injected with saline. Data are presented as means ± SEM for 6–8 animals. *p* > 0.05 vs. control group, nonsignificant (NS), unpaired two-tailed Mann–Whitney test, (GraphPad Prism 8.2).

Given that exogenous BK can increase brain trafficking of IV-infused leucocytes [21,24], we sought to examine whether the transient pharmacological opening of BBB by a B2R agonist can also allow the passage of circulating cancer cells to the brain. To this end, we combined the previously developed model of kinin-induced BBB disruption with a model of breast cancer brain metastases. Mice were infused intravenously with the B2R agonist NG291 (50 μg/kg over 5 min) or vehicle saline and simultaneously intracardiacally inoculated with 4T1-luc mammary cancer cells, which have a high propensity to form metastases, specifically in the brain [35,36]. Tumor burdens on histological brain slides 18 days following intracardiac injection were analyzed, and the results are presented in Figure 3. Figure 3A,B show typical H and E-stained histological brain slides from tumor-bearing mice in the two groups. Metastatic lesions were found widespread throughout the brain and consisted of cohesive, nest-forming neoplastic cells (Figure 3A,B inset). As shown in Figure 3C, the single-dose administration of NG291 did not significantly increase brain tumor burden (*p* = 0.6620), nor the number of metastases (*p* = 0.5237).

### 2.5. Effects of Kinin B2 Agonist Analog NG291 on BBB Permeability in the Irradiated Mouse Brain

**Figure 4:**
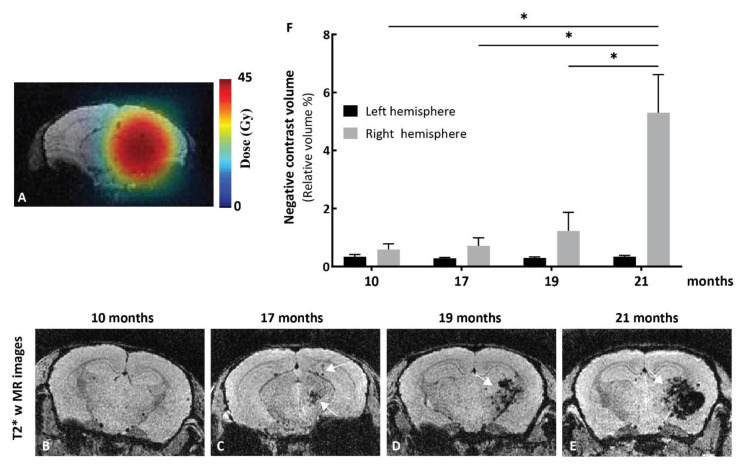
MRI studies in the mouse model of delayed cerebral radiation necrosis. (**A**) Treatment plan showing radiation contours superimposed on MR images of a mouse brain. The mouse brain right hemisphere was irradiated with a single radiation dose of 23.1 Gy at the 50% isodose (2.933 Gy/min; corresponding to a dose of 45 Gy at 100%). Pseudo-color bar indicates radiation dose. Representative T2*-weighted images of irradiated mice at (**B**) 10, (**C**) 17, (**D**) 19, and (**E**) 21 months after irradiation. The T2*-weighted sequence was used to detect necrosis, as necrosis results in the accumulation of hemoglobin residues (deoxyhemoglobin, hemosiderin, and ferritin) and T2*-weighted images are sensitive to the presence of those residues, which will produce negative contrast in the image. (**B**) At 10 months, T2*-weighted images show no sign of hemorrhage related to the presence of necrosis. (**C**) At 17 months, a small volume of negative contrast is observed in the region that received 100% of the radiation dose (white arrows). (**D–E**) This volume of negative contrast expands over time (white arrow) up to 21 months. (**F**) Negative contrast extracted from T2*-weighted images increases in the irradiated hemisphere. Bars indicate mean ± SEM (*n* = 3–4 animals). * *p* < 0.05 as compared to corresponding group, unpaired two-tailed Mann–Whitney test (GraphPad Prism 8.4).

**Figure 5:**
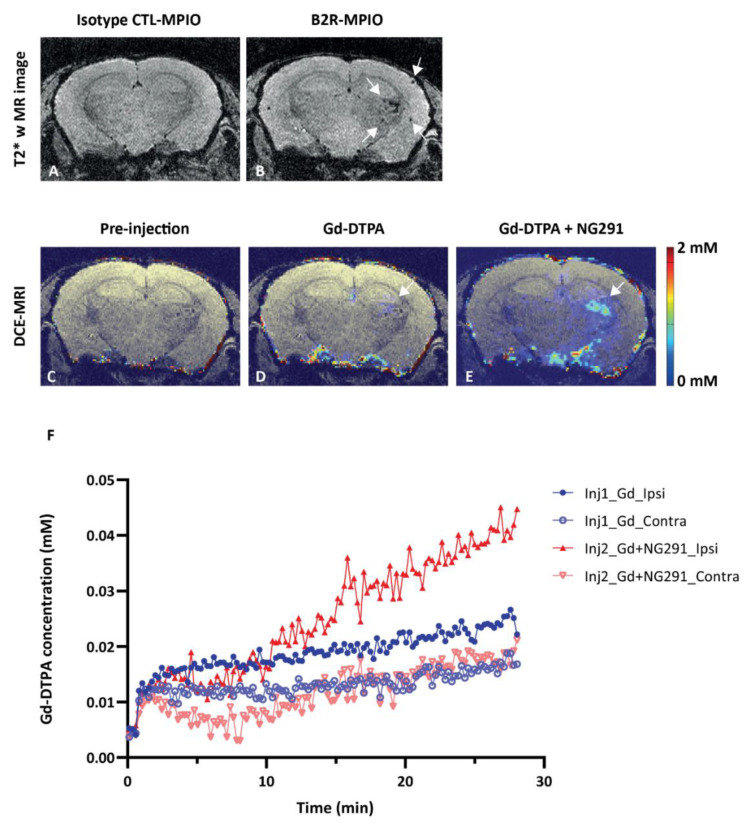
BBB modulatory effects of the B2R agonist analog NG291 on irradiated mouse brain. (**A**,**B**) Representative in vivo T2*-weighted 3D MR coronal image after (**A**) isotype control-microparticles of iron oxide (MPIO) or (B) B2R-MPIOs-injection. At 10 months after irradiation, specific retention of the contrast agent on activated vasculature is visible as signal voids (**B**, white arrow) in the right hemisphere, with little negative contrast observed in the contralateral control hemisphere or (**A**) with CTL-MPIO injection. (**C–E**) MRI assessment of changes in mouse brain vascular permeability after irradiation. Representative in vivo T1-weighted color-coded coronal images (**C**) before and (**D**) after Gd-DTPA injection and (**E**) after NG291 and Gd-DTPA. White arrows in D and E indicate the location of increased vascular permeability to Gd-DTPA. (**F**) Magnevist (Gd-DTPA) uptake in the irradiated (ipsilateral) and nonirradiated (contralateral) hemisphere as a function of time, before and after infusion of NG291. Signal increase in the irradiated hemisphere is at least 2-fold higher after B2R agonist injection. Pseudo-color bar indicates Gd-DTPA concentration.

Radiotherapy of brain cancer is known to induce acute and late pro-inflammatory reactions that involve the production of several cytokines (e.g., TNF-α, IL-1, IL-6, IL-8, IFN-γ) [37], many of which are potent inducers of kinin B1R/B2R expression [38,39]. Properties of B2R agonists can thus be distinguished in irradiated versus nonirradiated tumor-harboring brains. We begun by investigating B2R expression using MRI after radiation treatment of mice that received a single dose of 45 Gy in the right hemisphere of the brain (Figure 4A). A noninvasive MRI analysis of the mouse model of delayed radiation necrosis (Figure 4B–E, representative T2*-weighted images) revealed cerebral microvascular lesions related to radionecrosis at 17 months post-irradiation. Radiation-related lesions significantly increased in size over time in the irradiated hemisphere, as illustrated by the negative contrast quantification (Figure 4F). An anti-B2R antibody-labeled negative contrast agent (dark spots on T2*-weighted images) based on microparticles of iron oxide (B2R-MPIO) was injected 10 months after local irradiation, and the resulting T2*-weighted MR images are shown in Figure 5A–B. While minimal contrast variations were observed after administrations of isotype control antibody conjugated-MPIOs (Figure 5A, *n* = 4), evident hypointense signals restricted to the right irradiated hemisphere appeared on T2*-weighted images following B2R-MPIO injections (Figure 5B, white arrow, *n* = 4). The data showing specific binding and accumulation of the B2R-targeted MRI contrast agent on the cerebral vascular walls of irradiated tissues are strongly indicative of an upregulation of B2R at the cell-surface of microvascular endothelial cells.

**Figure 6:**
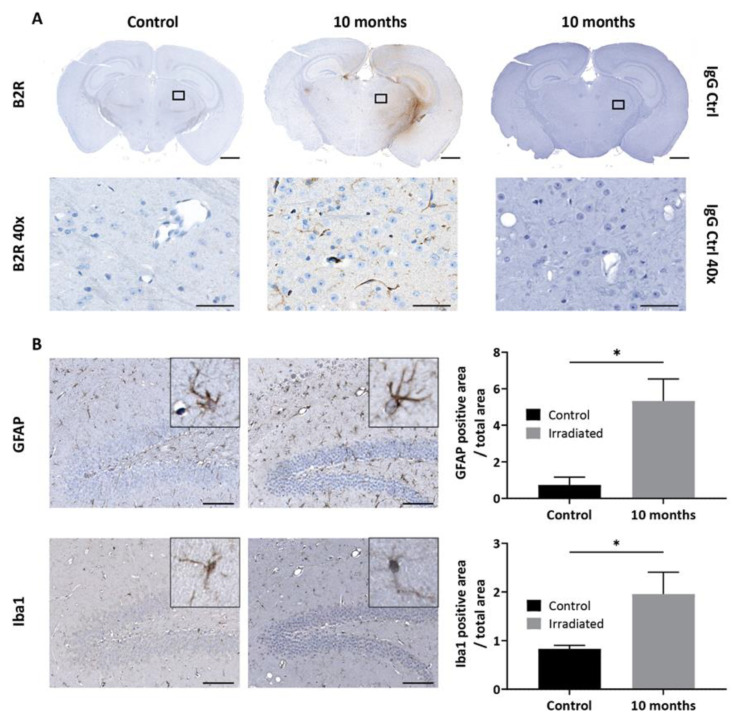
(**A**) Immunohistological detection of B2R expression in the mouse brain after irradiation. Minimal basal expression of B2R is detected in the brain of the control nonirradiated animal. Immunohistochemistry for B2R in a brain section of a representative animal at 10 months (middle) after irradiation confirms B2R expression in brain vessels (magnification 1× scale bar: 1 mm). No staining is observed with IgG antibody control. Images in the lower panel are high-resolution images of the area in the corresponding black rectangle (40× magnification, scale bar 50 µm). (**B**) Representative immunohistopathological images of glial activation after mouse brain irradiation. Nonirradiated (left panel) and irradiated (right panel) brain sections were stained with glial fibrillary acidic protein (GFAP) (astrocytes) or Iba1 (microglia)-targeted antibodies (magnification 20×; scale bar: 100 µm). Insets are high-resolution images showing cell morphology (magnification 80×). Bars indicate mean ± SEM (*n* = 4 animals per group). * *p* < 0.05 as compared to control group, unpaired one-tailed Mann–Whitney test (GraphPad Prism 8.4).

Having demonstrated that B2R is overexpressed at 10 months after brain irradiation, we proceeded with BBB disruption experiments. Dynamic contrast-enhanced (DCE)-MRI with Magnevist (Gd-DTPA) was used to assess the extent of BBB permeability following NG291 B2R agonist injection. Increased BBB permeability is revealed by hyperintense areas on T1-weighted images caused by extravasation and accumulation of Gd-DTPA, which does not cross the healthy BBB. Representative T1-weighted images of irradiated mouse brain are shown in Figure 5D–F. As expected, there was no contrast enhancement observed before Gd-DTPA administration (Figure 5C—the observed color results from noise at the scalp/brain interface). Minimal contrast enhancement was observed in the irradiated hemisphere after the first injection of Gd-DTPA (Figure 5D, white arrow). Contrast enhancement in the irradiated hemisphere was much stronger after NG291 and Gd-DTPA co-injection (Figure 5E, white arrow). The signal enhancement in the irradiated area reflects increased BBB permeability where B2R expression was detected on T2*-weighted images. Contrast enhancement was associated with an increase in Gd-DTPA concentration in the irradiated hemisphere (Figure 5F). After NG291 injection, there was a twofold increase in Gd-DTPA concentration in the irradiated hemisphere as compared to Gd-DTPA-alone injection and a fourfold increase compared to the contralateral hemisphere. Imaging results were confirmed by immunohistological analysis (Figure 6). While basal B2R expression is observed in control nonirradiated brains (Figure 6A, left panel), a strong endothelial B2R expression was shown in the right hemisphere of irradiated mouse brains (Figure 6A, middle). This fully corroborates the imaging data obtained with the endovascular B2R-MPIO MRI probe targeting B2R (Figure 5B). Immunoreactivity was barely visible in irradiated brain sections incubated with control IgG antibody (Figure 6A, right panel). Irradiation of the brain results in many other neuroinflammatory processes, as evidenced in Figure 6B. Indeed, compared to the control nonirradiated brain (left), glial activation was observed at 10 months after irradiation (right) with significantly increased numbers of glial fibrillary acidic protein (GFAP)-positive astrocytes and Iba1-positive microglial cells.

## 3. Discussion

In the present study, we were able to demonstrate (1) the ability of kinin peptide B1R and B2R agonist analogs (NG29 and NG291) to improve BBB penetration of the chemotherapeutic agent DOX in rats with moderately developed F98 glioblastoma (GBM); and (2) the efficacy of B2R agonist NG291 to induce BBB opening and extravasation of HMW dextran tracers into the brain of normal mice, using intraarterial (i.a) and i.v. delivery routes, respectively (Figure 1 and Figure 2). These results are similar to what has been found with Labradimil [2,13] and suggest that the BTB/BBB-permeabilizing properties of the B2R agonist NG291 could be applied to various drugs (or drug formulations), with a broad range of sizes (0.5 kDa to 2 MDa), which are unable to cross the BBB on their own. One example of this could be the association of kinin agonists with the recombinant monoclonal antibody Cetuximab (Erbitus; 152 kDa and 15 nm diameter; likewise, the 155 kDa TRIC-dextrans) targeting the EGFR that is overexpressed in more than 50% of patients with GBM. Despite being effective in the treatment of solid cancers outside the CNS, this compound failed to demonstrate clinical efficacy either as a single agent or in combination for GBM due to possible hindrance in the antibody’s ability to cross the BBB [40]. Support of this proposition is provided by the promising initial findings made from a recent prospective Phase I study showing the safety and efficacy of the mannitol-induced BBB disruption procedure prior to infusion of Cetuximab in EGFR-positive glioma patients [41]. Another possible therapeutic approach that should be envisioned for the future is the combination of kinin analogs with nanoparticles (NPs comparable in size of the 2 MDa FITC-dextrans) capable of delivering high, sustained doses of therapeutic agents into brain tumors while circumventing BBB/BTB active drug efflux mechanisms (ex. P-glycoproteins, multidrug resistance-associated proteins). This could be achieved following co-administration of drug-loaded NPs with free forms of kinin B1R or B2R agonist or with kinins-functionalized (-liganded) NPs containing drugs. These are ongoing studies in our laboratories and some reports on this have been published [31,32,42,43,44].

Regarding the mechanisms of passage of drug molecules across the BBB, there is ample evidence suggesting that B2R (and B1R) agonists, like the osmotic agent mannitol, enhance BBB/BTB permeability majorly through the endothelial paracellular pathway, i.e., increased diameters of intercellular clefts [13,19,45]. Interestingly, further studies showed that BK may increase BTB permeability, as well by means of the transcellular pathway, i.e., increased numbers of pinocytotic vesicles or caveolae [2,46,47]. The strategy exploiting targeted stimulation of the kinin receptors could perhaps assure more efficient brain drug delivery compared to those employing either one of the two modes of transport across the BBB. Comparative effectiveness studies are needed to confirm if there is such an advantage.

Our group and others have reported strong kinin B1R and B2R expression in glioma cell lines and human primary glioma samples [19,48,49], which could play roles in tumor growth and invasion [49,50,51,52]. This could raise a potential safety issue for the clinical applications of kinin agonists. However, the available evidence from preclinical studies do not lend support to an increased risk of accelerated growth and progression of GBM in animals receiving punctual, limited doses of synthetic kinin B1R and B2R agonists [13,19]. While not precluding potential roles of sustained endogenously produced kinins on breast cancer growth and metastasis, this also appears to hold true for the incidence of secondary brain tumors originating from this organ (Figure 3). In fact, our data point toward the BBB/BTB as the primary site of action for systemically administered kinin agonists in stimulating increased vascular permeability of water-soluble drugs. Moreover, the synthetic kinins are not proposed to be used alone but could rather serve as peptide adjuvants, i.e., in combination with other potent anticancer agents with well-established functions (and efficacy) for the treatment of brain tumors. We believe that the cytotoxic effects of these drugs will surpass the potential growth effects (if any) of systemically administered kinin analogs.

As mentioned earlier, Labradimil has been extensively tested in the clinic. Whereas initial studies co-infusing Labradimil and carboplatin intra-arterially showed promising results in terms of safety and efficacy [16], this therapeutic strategy was largely abandoned after two Phase II clinical trials failed to show any effect [14,15]. In fact, the authors of these studies chose to administer both compounds, Labradimil and carboplatin, intravenously. Furthermore, and somewhat incomprehensibly, they administered the carboplatin prior to the Labradimil infusion, potentially decreasing the impact of breaching the BBB given the lower bioavailability of carboplatin at that time. In our view, these study design choices may have ablated the chance to gaining adequate build-up in carboplatin concentration reaching the tumor cells. We believe that an intra-arterial cerebral infusion of carboplatin immediately after BBB permeation with Labradimil would be advised to better assess the potential efficacy of this latter class of compounds and increase success probability for the treatment of brain cancers.

The idea of using a mono or dual B1R/B2R agonist approach could further improve the extent of drug delivery beyond the BBB, as supported by data presented herein (Figure 1) and from previous reports [18,19,20]. This would enable an increased delivery compared to a single intra-arterial administration of Carboplatin (or other suitable chemotherapeutics), without requiring the heavy setup needed for hyperosmolar BBB disruption maneuvers that includes general anesthesia. Drug selection and formulations could also be improved considering the well-recognized concept of inter- and intra-tumor heterogeneity, a major contributor to drug resistance [53]. We perform i.c. chemotherapy infusion on a routine basis in our Sherbrooke hospital center (eight patients/week) in a controlled setting where we monitor the neurological conditions as well as vital signs of the patients, and we successfully treat patients every 4 weeks up to 16 cycles [7,8,54,55]. The addition of an infusion of a B2R or dual B1R/B2R agonist prior to chemotherapy infusion would represent a minor modification to our procedure. As recently pointed out by D’Amico et al. [56], i.c. delivery in conjunction with BBB disruption confers advantages notably in allowing delivery of a variety of agents directly to the targeted region, at more effective doses, while potentially sparing the body of systemic side effects.

Radiation therapy is part of the standard treatment paradigm for CNS tumors [57]. The short-term effects of prior radiotherapy on the BBB opening induced by Labradimil have been investigated in normal and focal-irradiated dog brains [58]. Results showed that a single, early i.v. injection of Labradimil (2–5 weeks after irradiation) remained effective at increasing permeability of already damaged BBB while not exacerbating the vasogenic edema associated with focal radiation injury. In this study, we focused on late post-radiotherapy complications leading to radionecrosis (Figure 4), which can manifest months to years in patients receiving radiation therapy, depending on the dose received. The selected irradiation dose of 45 Gy was well-tolerated by the animals, and no apparent signs of cognitive impairment was observed, although no specific test was performed to confirm this. Moreover, our DCE-MRI data provided strong evidence for the occurrence of enhanced basal BBB permeability in the early processes of radionecrosis, which can further be augmented upon acute pharmacological treatment with the B2R agonist NG291 (Figure 5). The observed vascular hyperresponsiveness to the B2R agonist is most likely attributed to the increased endothelial expression of B2R at tissue injury sites linked to inflammation (Figure 5 and Figure 6). It is noteworthy that both T1- and T2*-weighted MRI revealed a contrast by strategies based on B2R targeting (ex. B2R agonist analog, anti-B2R antibody-labeled iron-oxide NPs), improving the visualization of early stages of radionecrosis. Our findings are in line with the known contribution of BK in delayed brain damage after injury [59]. As more information becomes available on the exact causal links between cerebrovascular inflammation, BBB permeability, and neuroinflammation all leading to an accelerated cognitive decline [57], it may prove useful to increase the delivery of MRI contrast agents and therapeutic drugs to improve early detection and/or symptomatology of the disease. Indeed, MRI is commonly used to investigate radionecrosis but possesses low sensitivity and specificity. Therefore, invasive biopsy sampling remains the essential diagnostic tool for differentiating radiation necrosis from tumor recurrence [60]. Complementary studies will be necessary to evaluate the effects of kinin agonists on the BBB/BTB in regional tumor recurrence.

## 4. Materials and Methods

### 4.1. Synthesis of Kinin Peptide Analogs

Fmoc-amino acids used for the synthesis of peptides were obtained from Matrix Innovation (Québec, Canada), NeoMPS Inc. (Strasbourg, France) or Chem-Impex International (Wood Dale, IL, USA). Peptides were assembled on solid support by an automated Symphony-X peptide synthesizer (Protein Technologies Inc., Tucson, AZ, USA) using the 9-fluorenylmethyoxy-carbonyl (Fmoc) strategy and HATU/OxymaPure/diisopropylethylamine activation, as we described [25]. Thereafter, crude peptides were purified by analytical RP-HPLC (Waters 2535 module) on a C18 column (ACME C18, 10 µm, 250 × 30 mm, Canadian Life Science, Peterborough, Canada) using absorbance at 214 nm. The purity and identity of purified peptides were evaluated using ultraperformance liquid chromatography-tandem mass spectrometry (UPLC-UV-MS, Waters AQUITY-H-Class-SQD2, column Waters BEH C18 (1.7 µm, 2.1 × 50 mm)). Purified peptide fractions were pooled, lyophilized, and stored at –20 °C. Stock solutions (10 mM) of peptides were also prepared in Nanopure water and then stored at –20 °C until use. For in vivo studies, the stock solutions were diluted in sterile 0.9% saline before each experiment. Abbreviations of unusual amino acids employed for the peptide synthesis are described as follows: Hyp, trans-4-hydroxy-l-proline; Igl, 2-indanylglycine; Oic, l-[(3a5,7a5)]octahydroindol-2-yl-carboxylic acid; Thi, β-(2-thienyl)-l-alanine; ^N^Chg, N-cyclohexyl-glycine. The peptide RMP-7 was supplied by Bachem Bioscience (Philadelphia, PA, USA).

### 4.2. Cell Cultures

The F98 rat glioblastoma cell line (#CRL-2397) was purchased from American Type Culture Collection (ATCC, Manassas, VA, USA). 4T1-Luc cells (RRID: CVCL_J239) expressing luciferase were kindly provided by Theoharis C. Theoharides (Tufts University School of Medicine, Tufts Medical Center, Boston, MA, USA). F98, and 4T1-Luc and stable B2R-expressing HEK 293 cells were grown in Dulbecco’s modified Eagle’s medium (DMEM, Wisent, #319-015-CL) and in RPMI-1640 medium (Wisent, #350-000-CL), respectively, both supplemented with 10% fetal bovine serum (Wisent, #080-450) and penicillin (100 units/mL)/streptomycin (100 µg/mL) solution in noncoated flasks. Cultures were maintained in a humidified atmosphere of 5% CO2 and 95% air at 37 °C, and the medium was changed every 2–3 days.

### 4.3. Generation of Stable Human Kinin B2R-HEK 293 Cell Line

The pCDH-BDKRB2 vector was produced from the plasmid pcDNA3.1/human BDKRB2 by PCR amplification using the primers 5′-TCCATAGAAGATTCTAGAATGTTCTCTCCC-3′ and 5′-CTTCGCGGCCGCGGATCCTCACTGTCTGCT-3′, and introduced into pCDH-CMV-MCS-EF1 vector using BamHI et XbaI restriction sites. Lentiviruses were produced from pCDH-BDKRB2 vector in HEK293T cells using the X-tremeGENE HP transfection reagent (Millipore-Sigma, Oakville, ON, Canada). Lentiviruses were harvested 48 h post-transfection and used to infect QBI HEK 293A cells (ATCC). Transduced cells were selected using 5 µg/mL of puromycin (Thermo Fisher, Burlington, On, Canada). B2R mRNA expression was confirmed by qPCR assays (data not shown), and radioligand binding studies confirmed specific binding of [^3^H]-BK on live adherent cells (Kd = 3.8 nM, Bmax = 3.7 pmol/10^6^ cells).

### 4.4. Radioligand Binding Assays

Radioligand displacement binding assays were performed as we previously described [25], with slight modifications. HEK293 cells stably expressing human B2R were used for these assays (see text above). Cells were grown in 24-well plates and incubated with 1 nM [^3^H]BK (PerkinElmer # NET706250UC, Woodbridge, ON, Canada) per well in serum-free DMEM for 1.5 h at 4 °C in the absence or presence of increasing concentrations of competitors (10^11^ – 10^–5^ M). Radioactivity of samples was measured by a β-scintillation counter (PerkinElmer, Woodbridge, On, Canada). Binding affinities of peptides were expressed in terms of IC_50_ values—the molar concentration of an unlabeled agonist causing 50% displacement of specific binding (GraphPad Prism 8.2).

### 4.5. IP-One Accumulation Assays

The IP-One assay was performed on living, adherent human B2R-HEK293 cells according to the manufacturer’s recommendations (Cisbio, #62IPAPEB, Bedford, MA, USA). Briefly, 15,000 cells per well (384 shallow well plates) placed in FBS-free stimulation buffer (10 mM Hepes, 1 mM CaCl_2_, 0.5 mM MgCl_2_, 4.2 mM KCl, 146 mM NaCl, 5.5 mM glucose, 50 mM LiCl pH = 7.4) were treated with increasing concentrations of BK analogs (10^11^ – 10^–5^ M) for 30 min at room temperature. IP1-d2 and anti-IP1-Cryptate were added for at least 1 h. Fluorescence signal was measured using a Tecan M1000 plate reader (excitation at 320 nm and emission at 620 and 665 nm). EC_50_ values were calculated by curve-fitting using GraphPad Prism 8.2. 

### 4.6. Animal Studies

Experiments were performed with male Fischer rats (body weight 230–250 g, Charles River Laboratories) and female BALB/c mice (18–22 g, Charles River Laboratories). Animals were maintained under standard diurnal conditions and were allowed access to food and water ad libitum. Animal experiments were approved by the Institutional Animal Care and Use Committee of the Université de Sherbrooke and performed in accordance with the Canadian Council on Animal Care guidelines.

#### 4.6.1. F98 Syngeneic Rat Model of Glioblastoma

Fischer rats were surgically inoculated with 1 × 10^4^ F98 cells (in 5 μL) in the region of the right caudate nucleus, as previously described [18,19,20]. Tumors were allowed to grow for 10 days, corresponding to a midstage tumor development.

#### 4.6.2. Doxorubicin Delivery in Rat Brain

F98-bearing rats were anesthetized with a mixture of ketamine: Xylazine (87:13 mg/kg, intraperitoneal), and the external carotid artery was catheterized in a retrograde fashion using PE-50 intramedic tubing such that the tip of the catheter lay just above the bifurcation. For the BBB permeabilization procedure, the animals received a single intracarotid infusion of vehicle (sterile 0.9% saline), B1R agonist (NG29; 50 nmol/kg/min for 5 min), B2R agonist (NG291) (10 nmol/kg/min for 5 min), or a combination of NG29 and NG291. Immediately after the end of the BBB disruption procedure, doxorubicin (4 mg/kg; 500 μL, 5 min) (Pfizer, 2 mg/mL stock; provided by the Pharmacy Department of the CHUS (Canada)) was infused via the same cannula. Total volume infused was kept constant at 1.0 mL. One hour after the end of the infusion, anesthetized rats were euthanized by transection of the vena cava followed by an intra-cardiac injection of saline (200 mL) in order to flush the blood from the brain. The brain was rapidly removed and placed in physiological saline. The tumors, as well as size-related samples of peritumoral tissue and matched tissue located in the contralateral hemisphere, were resected. These samples were weighed (~30–50 mg/sample) and soaked in acidified ethanol (50% ethanol in 0.3 N HCl). After homogenization with a tissue blender (IKA works Inc., Wilmington, NC, USA) and refrigeration for 24 h at 4 °C, the samples were centrifuged at 16,000× *g* for 25 min. Supernatants were collected, concentrated using a Speed-Vac (SC 110A Concentrator, Thermo Savant, Holbrook, NY, USA), and resuspended in 100 μL of acidified ethanol. The fluorescent intensity of the sample (100 μL each) was measured in a 96-well microplate using a benchtop fluorometer (Infinite M1000; Tecan Group Ltd., Mannedorf, Switzerland; Ex/Em: 480/590 nm). The concentration of DOX was determined from a standard calibration curve derived from twelve serial concentrations (1 ng–0.1 mg/100 μL) (Graph Prism 8.2). For illustrative purposes, biodistribution of DOX in brain tumors was also assessed in some of the above experimental groups (untreated control, vehicle saline + DOX or B1/B2 agonists + DOX) using confocal microscopy. Then, 24 h after treatments, animals were euthanized and exsanguinated under anesthesia, as mentioned above. Thereafter, brains were harvested, embedded in OCT, and flash-frozen in liquid nitrogen. Frozen tissues were kept at –80 °C until further processing. Brain tissues were cut on a cryostat in 20 µm-thick coronal sections, and the tissue slices were thaw-mounted onto surface-treated glass slides (Superfrost plus, ThermoFisher Scientific, Burlington, ON, Canada). The fluorescence characteristics of DOX were used to directly monitor localization of this drug without utilizing additional dye.

#### 4.6.3. Cranial Window Surgery

Mice were anesthetized with an intraperitoneal injection of ketamine/xylazine (87/13 mg/kg). Before surgery, the level of anesthesia was assessed through a paw pinch and the top of their head was shaved. Mice were then placed on a stereotactic frame and their eyes covered with eye-protecting gel. An incision was made over the skin of the right hemisphere and the remaining epidermis was removed with a cotton swab soaked with isopropyl alcohol. A 2 mm-diameter circular craniotomy (coordinates: 2 mm posterior, 2 mm lateral from the bregma) was carefully drilled while avoiding damage to the underlying dura using a dental drill. To reduce heat damage, inflammation, and control bleeding, drilling was frequently paused, and saline was applied to cool the skull. A margin of the bone was created around the craniotomy allowing the central skull piece to be released and, under a drop of saline, carefully separated from the skull using forceps. Gelfoam previously soaked in saline could be applied occasionally to stop any small bleeding that could occur. The exposed brain was covered with saline followed with a 5 mm diameter (0.16–0.19 mm thick) round glass coverslip (World Precision Instruments, Sarasota, FL, USA). The edges of the coverslip were glued to the skull surrounding the window using cyanoacrylate glue (Lepage, Canada). After the glue had dried completely, the edges were further secured with dental cement (Teets Denture Material). All the exposed skull and wound edges were also covered with dental cement. Mice were then allowed to recover on a heating pad until fully mobile and returned to their cages. Intravital microscopy was conducted 2 weeks after surgery to minimize inflammation related to surgical manipulations.

#### 4.6.4. Intravital Fluorescence Microscopy

Mice were imaged through the cranial window using an inverted confocal laser scanning microscope (FV1000, Olympus, Japan) operated with Olympus FluoView software version 1.6a. To image the vasculature, a catheter was placed in the tail vein and secured with a tape for the injection of fluorescent dextran and drug infusion. The mouse was held in the supine position under a UPLFLN ×10/0.30 NA objective (working distance of 10 mm) using a customized plate designed to fit the microscope motorized XY stage. The animal and microscope stage were rotated to expose the cranial window and cortical surface. The stage was covered by a temperature-controlled chamber, allowing the mouse body temperature to be maintained during the experiment. A time lapse video of the vasculature of the mouse cortex was recorded (frame interval ~ 2.7 s, 800 × 600 pixels) upon intravenous administration of dextran conjugated with fluorescein isothiocyanate (FITC) or tetramethylrhodamine (TRITC) of 2 MDa (FD2000S, Sigma) or 155 kDa (T1287, Sigma) in size, respectively (10 mg/mL dissolved in PBS; 40 mg/kg). Then, a stack of XY scans (field of view (FOV) = 1600 × 1200 pixels), every 14 µm step in the axial direction for a 518–686 µm total depth of the cortex, was acquired (voxel size = 0.9 × 0.9 × 14 µm^3^; resolution = 1.111 pixel/µm; 4 µs/pixel; scan time = 355–477 s depending on the total cortical depth imaged). The images stack allowed the integrity of the vasculature to be examined. One location within the cranial window was selected, and time series scans were acquired at a single transverse plane at varying depths with the following parameters: FOV = 1024 × 768 pixels; voxel size = 0.937 × 0.937 × 28 µm^3^; resolution = 1.0672 pixel/µm; 2 µs/pixel, total scan time = 30 min. The B2R agonist NG291 for BBB disruption (50 µg/kg) or vehicle (isotonic saline) in the control condition was infused intravenously 4 min after the start of the scan. 

#### 4.6.5. T1 Syngeneic Mouse Model of Brain Metastasis

Mice were anesthetized using 1.5 L/min oxygen-containing isoflurane (3% for induction, 2% for maintenance) and secured with tape in a supine position on a heating pad. A total of 1 × 10^5^ 4T1-Luc cells suspended in 100 µL of sterile saline was slowly injected into the left ventricle of the heart using a 1 mL Hamilton syringe mounted with a 30G needle as described previously [61]. For BBB disruption experiments, a cannula was placed in the caudal vein and a single dose of NG291 (50 µg/kg/5 min) was infused into the caudal vein while simultaneously injecting the cancer cells intracardially. Successful intracardiac injection and tumor development was evaluated weekly by bioluminescence imaging. The development of brain metastatic tumor burden was determined by histological analysis.

#### 4.6.6. Mouse Model of Radiation-Induced Brain Injury

Female Balb/c mice were anesthetized using 1.5 L/min oxygen containing isoflurane (3% for induction, 2% for maintenance) and positioned on a custom-designed stereotactic bed [62] compatible with the Leksell Gamma Knife Perfexion (Elekta AB, Stockholm, Sweden). A maximum dose of 45 Gy (23.1 Gy at the 50% isodose) was delivered through 4 mm collimators in the right hemisphere of the brain. Mouse and human brains have different sensitivities to radiation. This dose was selected because it allows the development and slow progression of radiation necrosis (approximatively appearing at 17 months post-irradiation in T2-weighted MRI; Figure 4), similar to what may be observed in brain cancer patients who received radiotherapy [63]. After irradiation, mice were placed on a heating pad to recover, and were then returned to their cages.

##### Preparation of MPIOs

Microparticles of iron oxide (MPIO, size range 0.76–1.63 μm) with p-toluenesulfonyl reactive surface groups (Invitrogen, Carlsbad, CA, USA) were conjugated to purified polyclonal rabbit anti-mouse antibodies specific to the BK-B2R (OABF00968, Aviva Systems Biology, San Diego, CA, USA; B2R-MPIO) or normal rabbit IgG as a negative control (2729S, Cell Signaling Technology, Danvers, MA, USA; isotype control, CTL-MPIO), following a method described previously [64]. MRI acquisition was performed before and 3 h after i.v. injection (0.125 mg Fe in 100 μL PBS) of one of these conjugated MPIOs.

##### In Vivo MRI Acquisitions and Image Analysis

Experiments were conducted on a 210 mm small animal 7 Tesla scanner (Varian Inc, Palo Alto, CA, USA) with a dedicated mouse head coil (RAPID MR International, Columbus, OH, USA) before (control, *n* = 4) or 10 months (*n* = 4) after irradiation. T2*-weighted images were acquired using a three-dimensional (3D) gradient-echo sequence: TR = 50 ms, TE = 25 ms, flip angle = 15°, data matrix = 256 × 192 × 96, field of view (FOV) = 20 × 15 × 10 mm^3^, bandwidth = 26.2 kHz, 2 averages. The T2*-weighted sequence was used to visualize B2R expression in irradiated brains by imaging B2R with MPIOs-B2R.

For dynamic contrast-enhanced (DCE)-MRI imaging, pre-contrast T1-weighted imaging with an array of flip angles (α = 10°, 20°, 25°, 35°, 50°) was performed to acquire a T1 map with the following parameters: TR = 100 ms, TE = 2.79 ms, data matrix = 128 × 128, FOV = 20 × 20 mm^2^; 1 average; 10 slices; 1 mm slice thickness. Then, a dynamic acquisition with the same parameters and a fixed angle at 30° was acquired before, during, and after injection of contrast agent. A first bolus i.v. injection of Gd-DTPA (Magnevist, 142.9 mM; Berlex, QC) was administered 30 s after acquisition initiation (230 µL over 120 s) to determine basal BBB permeability. At the end of the dynamic sequence, a second imaging session was performed with NG291 (50 µg/kg) and Gd-DTPA co-injection (bolus of 280 µL injected over 120 s) to evaluate BBB permeabilization. For all MRI acquisition, animals were anesthetized using 1.5 L/min oxygen containing isoflurane (3% for induction, 2% for maintenance).

Negative contrast quantification (hypointensities) in T2*-weighted images was performed as previously described [61]. Briefly, a brain template and its segmented areas were registered (affine transform) to each data set using advanced normalization tools (ANTs, version 2.1.0). A threshold was determined for each axial slice as the mean signal minus four standard deviations, and negative contrast volume was determined as the total volume of voxels with a magnitude value under this threshold for each hemisphere.

##### Histological Analysis

Under deep anesthesia, mice were transcardially perfused with heparinized PBS followed by 4% paraformaldehyde (PFA) using a perfusion pump. Their brains were removed, postfixed for 24 h, and paraffin-embedded. Five-micrometer-thick longitudinal sections were mounted on silanized slides (VWR International, Edmonton, AB, CA) for either pathological change examination or immunohistochemical analysis.

Brain sections from mice inoculated with 4T1-Luc cells were stained with hematoxylin-eosin (H and E) for histological examination and metastases quantification using the NanoZoomer Digital Pathology software (Hamamatsu, NDP.view version 2.7.25). H and E staining was performed on brain sections every 20–30 µm. The relative total area and number of metastatic foci in the entire brain section were calculated from manual delineation of metastases in digital images of H and E-stained brain slides using the NanoZoomer NDP.view2 viewing software free hand tool. The relative area is the sum from all slides. A total of 68 slices were analyzed.

Immunohistochemistry (IHC) on sections from irradiated brains was performed as described elsewhere [61,65]. Brain tissues sections were incubated for 60 min with a blocking solution consisting of 2% bovine serum albumin (BSA) in Tris-buffered saline (TBS, pH 7.6), and then incubated overnight with the chicken anti-glial fibrillary acidic protein (GFAP, 1:200, AB5541, Sigma, Oakville, ON, CA) or the rabbit anti-ionized calcium binding adapter molecule-1 (Iba1, 1:1 000, 013–27691, Fujifilm Wako, Richmond, VA, USA) antibody. Antibody-bound sections were revealed using biotin-conjugated goat anti-chicken (1:200; Sigma, Oakville, ON, USA) or horseradish peroxidase-conjugated goat antirabbit (1:500; Jackson ImmunoResearch) secondary antibody and 3, 30-diaminobenzidine. An extra 1 h incubation step with the Vectastain ABC kit mixture (#PK-6101, Vector Laboratories, Burlingame, CA, USA) was added for section incubated with the anti-GFAP primary antibody. IHC staining of B2R was performed with an automated system (Dako Autostainer plus) using the Envision Flex High pH visualization system (Dako, Agilent Technologies, Mississauga, ON, CA) [19]. To this end, sections were stained with the rabbit polyclonal anti-B2R antisera AS277-83 (1:2000; kindly given by Dr. W. Müller-Esterl, University of Frankfurt Medical School, Germany) whose target specificity has been described elsewhere [66,67,68,69]. Negative controls (sections with isotype control antibody—normal goat IgG, AB-108-C, R&D Systems or normal rabbit IgG, 2729S, CST) were included to verify the specificity of immunostaining. Counterstaining was performed with hematoxylin. Additional sections were stained with hematoxylin–eosin (H and E) to assess general pathology. Sections were digitized and visualized using the Hamamatsu NanoZoomer 2.0-RS digital slide scanner (Hamamatsu Photonics) and the NanoZoomer Digital Pathology software (NDP.view2).

Quantification of GFAP and Iba1 staining from acquired images of the right cerebral hemisphere was performed using the open source ImageJ Fiji software (version 1.53c; https://imagej.nih.gov/ij/). For each animal, one brain section was analyzed. Stained cells were extracted by applying a threshold on IHC images and quantified on three random regions of interest (ROI) in the irradiated right hemisphere. Results are presented as the mean of the quantified cells in all ROI relative to the total area of those ROI.

## 5. Conclusions

In conclusion, our findings are consistent with those of other studies evaluating the efficacy of kinin analogs as reversible BBB permeabilizers for brain drug delivery in a variety of CNS disorders, and substantiate their value as selective pharmacological tools for studies on the physiology and pathophysiology of kinin B1R and B2R. They may also hopefully serve to revive an interest in the potential clinical applications of kinin analogs in CNS diseases.

## Figures and Tables

**Table 1:** Bioanalytical analysis and pharmacological characteristics of kinin B2 receptor (B2R) agonist analogs.

Sequence (Codename)	Theoretical m.w.	Observed m.w	Purity (%)	Binding (IC_50_; nM)	IP1 Assays (EC_50_; nM)	Refs
H-Arg^1^-Pro^2^-Pro^3^-Gly^4^-Phe^5^-Ser^6^-Pro^7^-Phe^8^-Arg^9^-OH (BK)	1060.2	1060.4	99.0	4	0.3	-
[Hyp^3^, Thi^5^, ^N^Chg^7^, Thi^8^]-BK (NG291)	1130.4	1130.5	100	3	0.9	[25]
[Hyp^3^, Thi^5^, (^4^Me)Tyr^8^(ΨCH_2_NH)Arg^9^]-BK (Labradimil)	1098.3	1098.3	99.8	17	47	[13]
dArg[Hyp^3^, Igl^5^, Oic^7^, Igl^8^]-BK (B9972)	1338.6	1339.4	99.3	199	158	[30]
d(retroinverso)-BK (RI-BK)	1060.2	1061.8	95.2	>10,000	>10,000	[31,32]

Molecular weight (m.w.) and purity of peptides were determined by HPLC-MS analyses. Binding and potency data are the mean results of two independent experiments performed in duplicates.

## Supplementary Materials

The following are available online at https://www.mdpi.com/1424-8247/13/10/279/s1. Videos S1 and S2: Video imaging of B2R agonist NG291-mediating BBB disruption and extravasation of FITC-dextrans (2 MDa; video S1) or of TRITC-dextrans (155 kDa; video S2) within brains of normal mice, as determined by real-time intravital microscopy.
